# Supplementary material for: Oxidative phosphorylation is a key feature of neonatal monocyte immunometabolism promoting myeloid differentiation after birth
Source: Nat Commun. 2025 Mar 6;16:2239. doi: 10.1038/s41467-025-57357-w (PMC11885822; doi:10.1038/s41467-025-57357-w)
Supplement: Supplementary file 1 — Supplementary Information [file 41467_2025_57357_MOESM1_ESM.pdf]

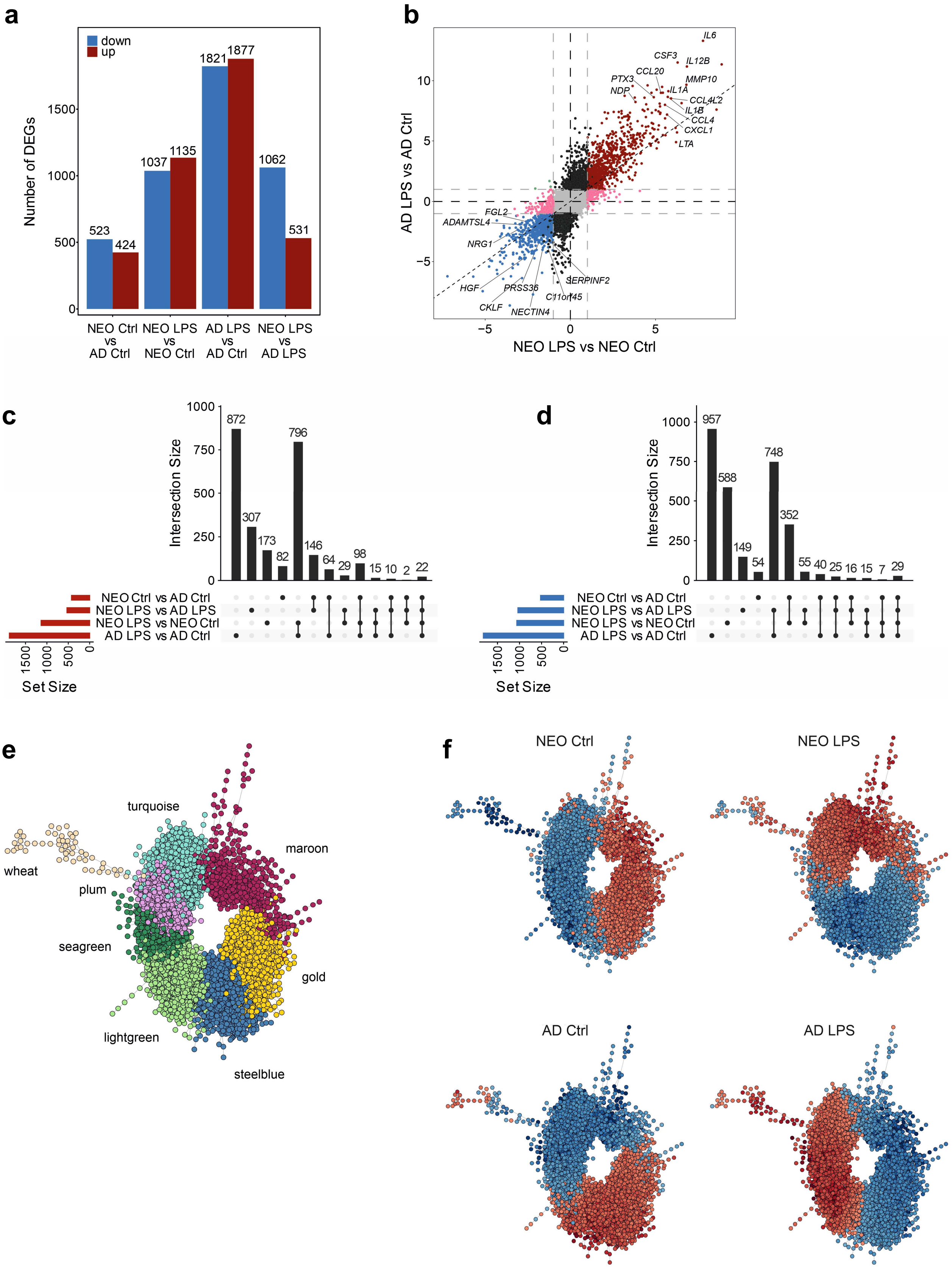

Supplementary Fig. 1. Network analysis using hCoCena reveals differentially expressed gene modules in NEO and AD Mo at baseline and after LPS activation. a Diagram indicating the numbers of differentially expressed genes (DEGs) between the respective conditions. **b** FC-FC plot depicting shared upregulated (red), shared downregulated (blue), DEGs uniquely regulated in NEO (pink) or AD (dark grey), as well as DEGs inversely regulated (green), and highlighting the top 15 up- and downregulated DEGs filtered for secretome. **c** UpSet plots of upregulated and **d** downregulated DEGs showing the overlap in the respective comparisons (FC > 2, ihw adjusted p < 0.05). **e** Integrated hCoCena network colored by module. **f** Integrated hCoCena networks colored by significant GFCs within the respective groups. Red, upregulated; blue downregulated.

a

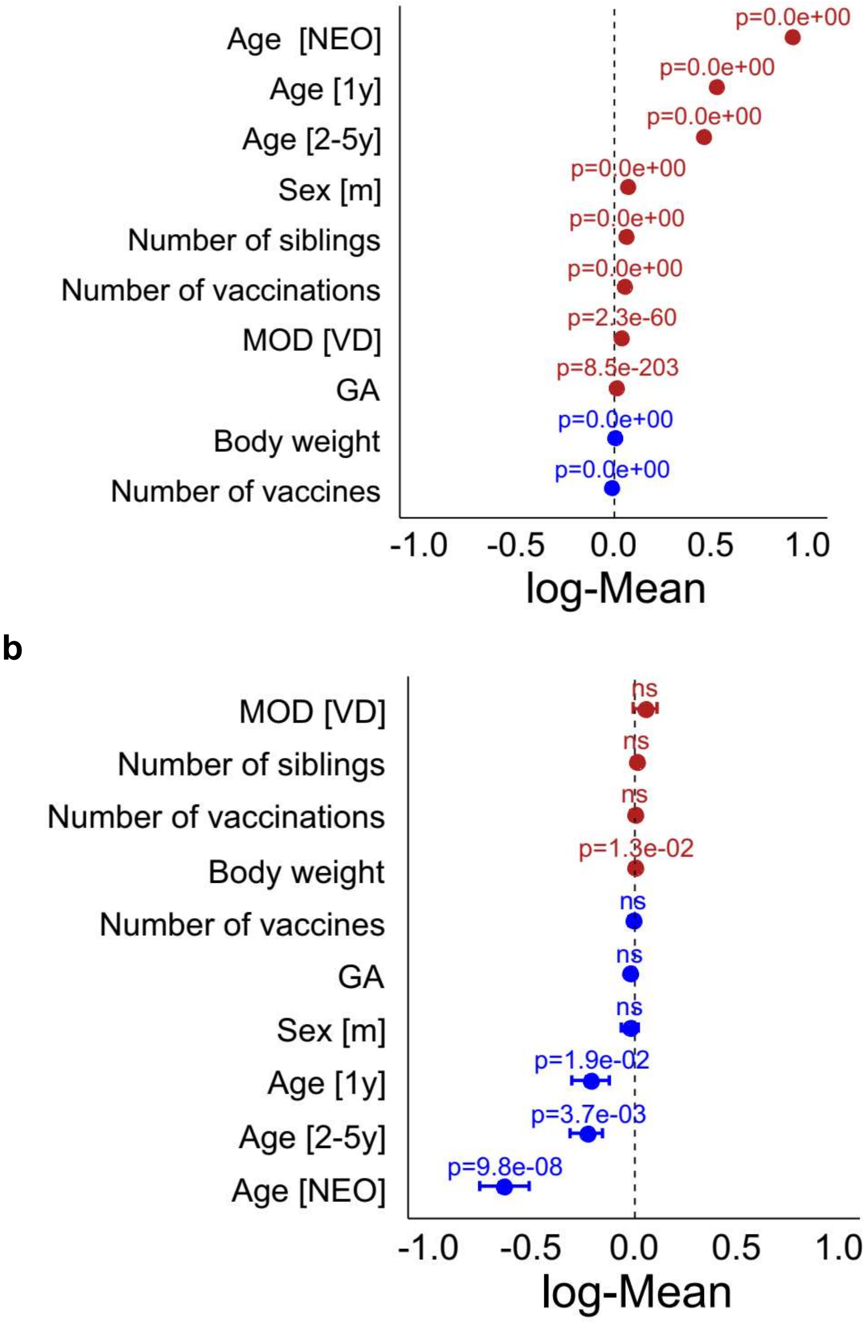

Supplementary Fig. 2. Impact of endogenous and environmental factors on the metabolic phenotype of Mo. Proband-specific indicated factors potentially influencing the metabolism of Mo from the study population (NEO: *n* = 10; 1y: *n* = 16; 2-5y: *n* = 8; AD: *n* = 15) were integrated with the metabolic analysis data to determine their effect sizes on the respective metabolic parameter by building a generalized linear model plotted as log means ± CI. **a** Effects on the energy metabolism (Puro MFI). **b** Effects on the glycolytic dependence. The indicated p-values were determined using the Wald test. GA, gestational age, MOD, mode of delivery; VD, vaginal delivery.

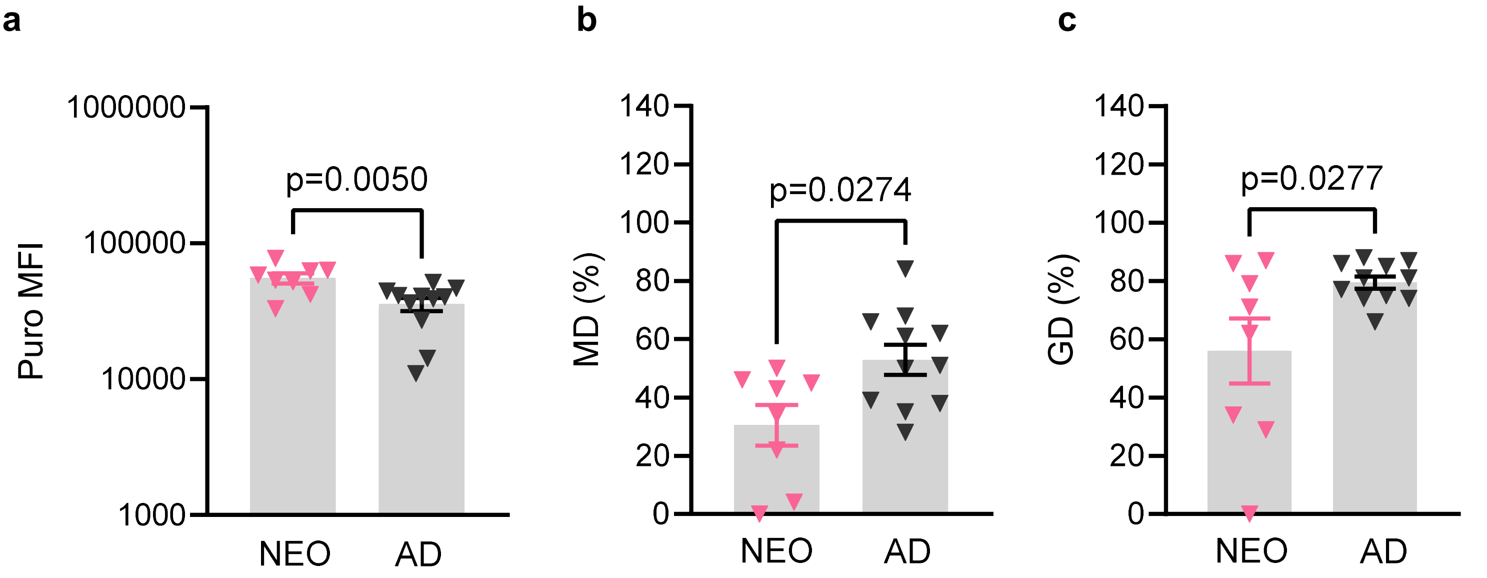

Supplementary Fig. 3. Metabolic differences between NEO and AD Mo after 24h of LPS activation. a-c Isolated NEO (n = 8) and AD (n = 11) MNCs were treated for 24h with 1 µg/ml LPS and subjected to SCENITH studies**.** Direct comparison of **a** energy metabolism (Puro MFI), **b** OXPHOS (MD) and **c** glycolytic dependence (GD) in LPS-treated NEO and AD Mo plotted as means ± SEM. p-values were determined using two-sided MWU-tests.

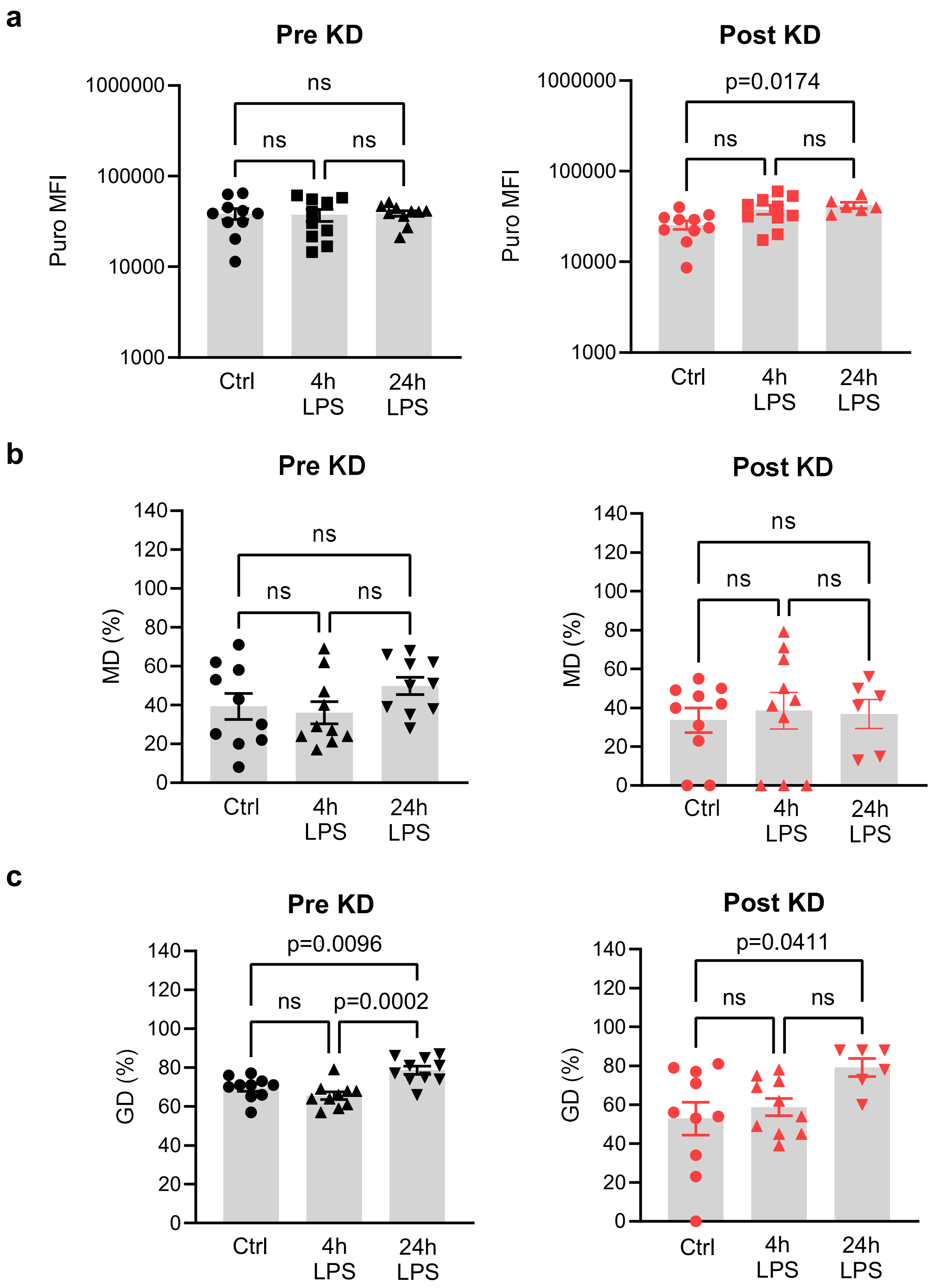

Supplementary Fig. 4. Metabolic profile of Mo during LPS activation before and after a ketogenic diet in adults. a Energy metabolism, **b** OXPHOS and **c** glycolytic dependence in Mo over time of LPS activation as assessed by SCENITH studies in MNCs from AD donors before (Pre-KD) and 7 days after (Post KD) start of ketogenic diet after treatment for 4h (each *n* = 10) and 24h (Pre-KD *n* = 10, Post KD *n* = 6) with 1 µg/ml LPS compared to baseline (untreated Ctrl; each *n* = 10). Plotted are means ± SEM. Significant differences were determined using one-way ANOVA and *post hoc* Tukey's multiple comparison tests.

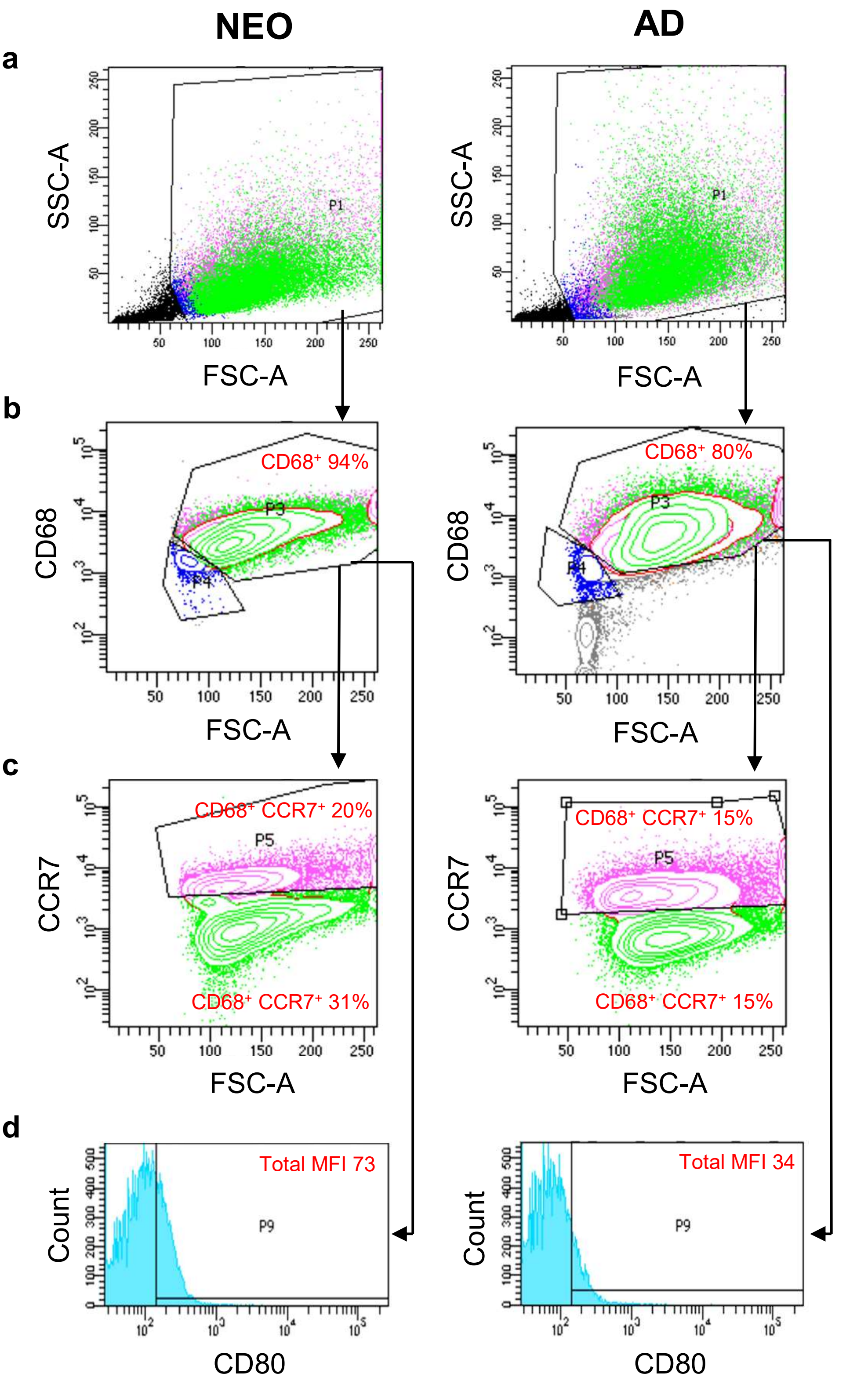

Supplementary Fig. 5. Flow cytometry analysis of NEO-MDM and AD-MDM. After 14 days of differentiation, NEO-MDM (left panels) and AD-MDM (right panels) were stained against CD68, CCR7 and CD80 and subjected to flow cytometry. Shown are representative plots from samples relating to Fig. 5h-j. After exclusion of dead cells and cell debris (**a**), cells were gated to determine the proportions of CD68+ MDM (**b**) and CD68$^+$ CCR7$^+$ and CD68$^+$ CCR7$^-$ MDM (**c**) from living cells (a) and the MFI of CD80 expression on CD68$^+$ MDM (**d**) according to the shown sequence.

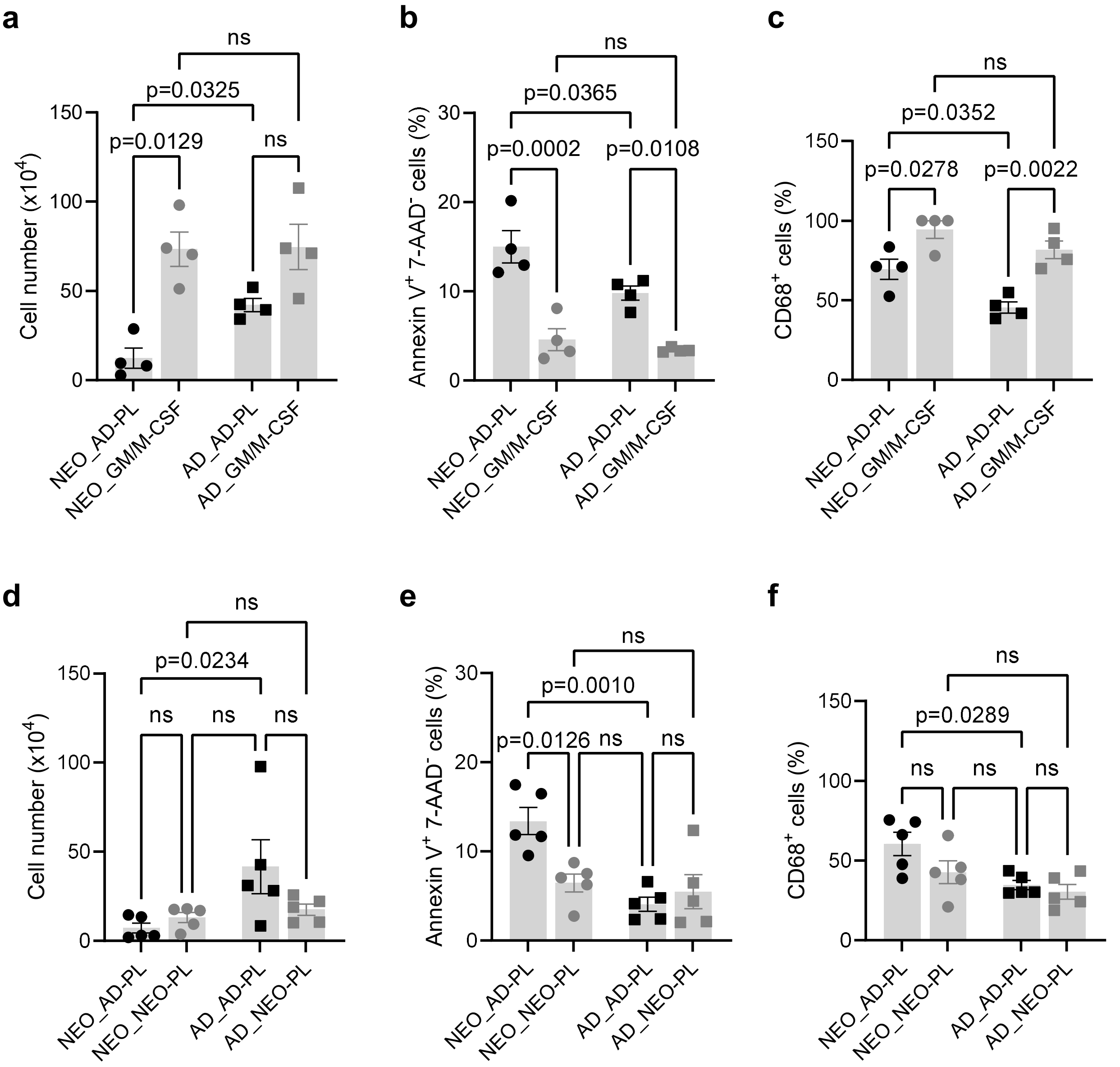

Supplementary Fig. 6. Impact of cytokine-driven polarization and cord blood plasma on the age signature of NEO and AD MDM differentiation. NEO and AD Mo were *ex vivo* differentiated for 7 days into MDM **a-c** in the presence of human AD blood plasma (AD-PL) or GM-CSF and M-CSF (GM/M-CSF) (each *n* = 4) or **d-f** in the presence of AD blood plasma (AD-PL) or cord blood plasma (NEO-PL) (each *n* = 5). **a,d** MDM numbers at harvest on d7. **b,e** Proportions of apoptotic cells (Annexin V$^+$7-AAD$^-$). **c,f** Proportions of CD68$^+$ MDM. Plotted are means ± SEM. Significant differences were determined using one-way ANOVA and *post hoc* Tukey's multiple comparison tests.

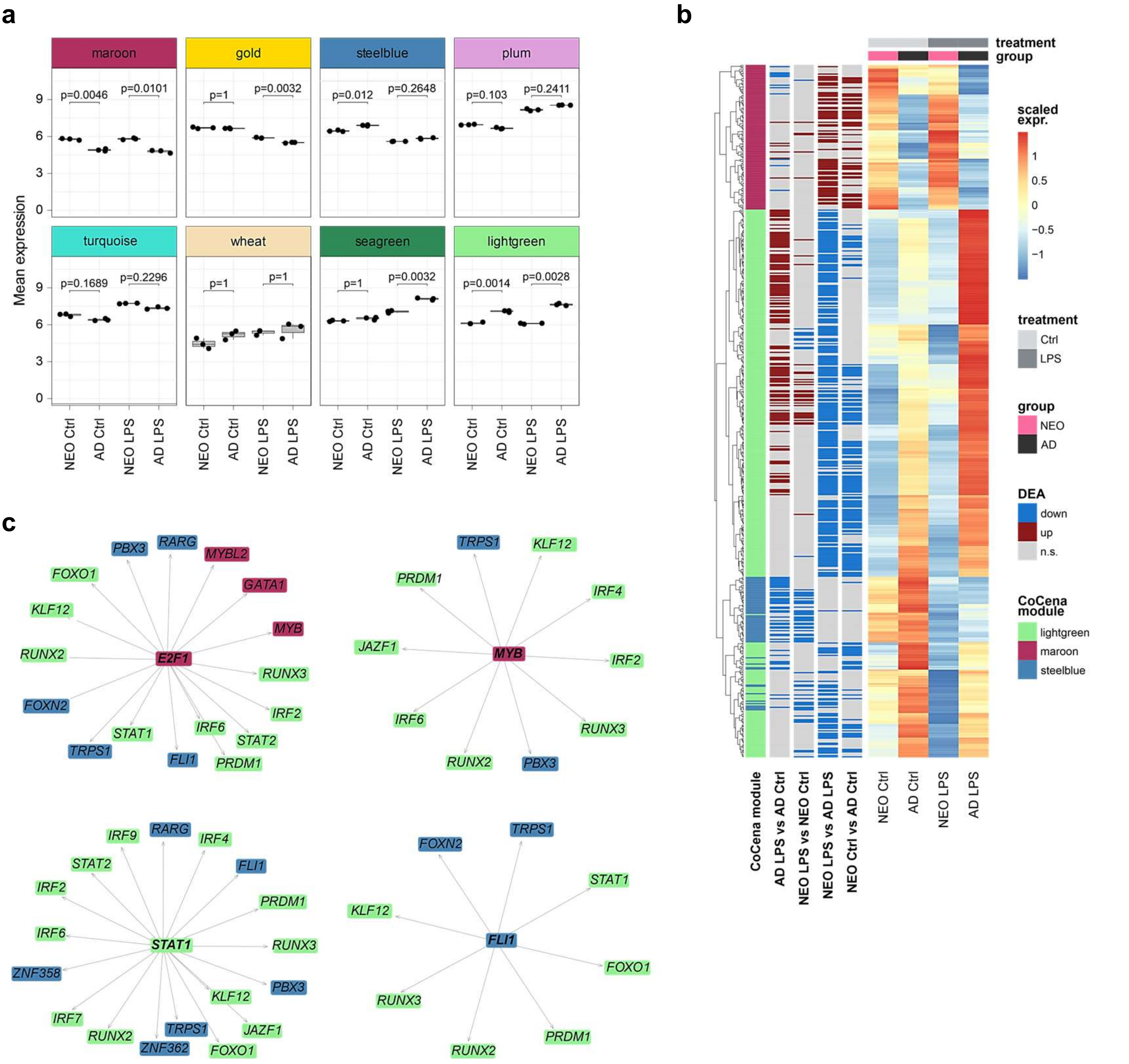

Supplementary Fig. 7. Candidate transcriptional regulators of the postnatal ontogeny of immunometabolism. a Mean expression of genes within the respective network modules in respective age and treatment groups. Box plots show medians (central line), interquartile ranges (box edges) and whiskers extending to the smallest and largest data points. The p-values were determined using unpaired two-sided t-tests. **b** Heatmap of the scaled mean expression of *E2F1* target genes in indicated groups. Genes are annotated by their network module affiliation and significant expression differences in the respective comparisons (red, upregulated, blue, downregulated). **c** Target TFs of *E2F1*, *MYB*, *STAT1*, and *FLI1*, module affiliation color-coded.

Supplementary Table 1. Clinical characteristics of the SCENITH study population.

	Newborn (NEO)	Infant (1y)	Toddler / Preschooler (2y-5y)	Adult (AD)
Number of probands	10	16	8	15
Age [years][A]	0 (0)[B]	0.5 (0.1)	3.3 (0.6)	29.8 (3.4)
Sex [female/male]	3 / 7	8 / 8	4 / 4	7 / 8
Mode of delivery [CS/VD]	9[C] / 1	8[D] / 8	2 / 6	1 / 14
Gestational age [weeks][A]	38.7 (0.3)	37.2 (1.2)	38.8 (1.0)	39.1 (0.5)
Birth weight [g][A]	3542 (130)	3144 (269)	3086 (259)	3333 (110)
Body weight [kg][A]	3.5 (0.1)	7.06 (0.6)	15.5 (2.2)	73.9 (3.9)
Previous pregnancies [0/ ≥1]	4 / 6	8 / 8	4 / 4	8 / 7
Number of siblings[A]	0.7 (0.2)	1.2 (0.4)	0.6 (0.3)	1.0 (0.2)
Number of vaccinations[A]	0 (0)	2.4 (0.5)	7.1 (0.8)	17.0 (1.8)
Number of total vaccines[A]	0 (0)	14.1 (2.3)	32.4 (1.7)	41.5 (3.2)

[A]Mean (± SEM); [B]CB, cord blood; CS, cesarean section; VD, vaginal delivery; [C]7 primary CS, 2 secondary CS; [D]7 primary CS, 1 secondary CS

Supplementary Table 2. Characteristics of the KD study population.

	Pre KD[B]	Post KD[C]
Number of probands	10	10
Age [years][A]	30.8 (4.5)	30.8 (4.5)
Sex [female/male]	5 / 5	5 / 5
Urine ketones [mg/dL][A]	0 (0)	57 (14)
Fasting urine glucose [mg/dL][A]	0 (0)	0 (0)
Body weight [kg][A]	71 (5)	70 (5)
BMI [kg/m^2][A]	23 (1)	23 (1)

[A]Mean (± SEM); [B]2 days before start of KD; [C]day 7 after start of KD; KD, ketogenic diet; BMI, body mass index

Supplementary Table 3. Age-appropriate dietary reference intakes.

	Neonate (Birth-3m) - Breast milk[A]	Infant (4m-1y)	Toddler / Preschooler (2y-5y)	Adult - Standard European diet[B]
Total calories [kcal/kg/d]	115	105	95	50 - 60
Carbohydrates (% of kcal)	42	45 - 60	45 - 60	45 - 60
Fat (% of kcal)	51	35 - 40	35 - 40	20 - 35
Protein (% of kcal)	7	5 - 20	5 - 20	5 - 20

[A]https://doi.org/10.1016/B978-0-12-815350-5.00007-3; [B]Dietary reference values for the EU population as derived by the EFSA Panel 2017

Supplementary Table 4. List of candidate transcriptional regulators of the postnatal ontogeny of immunometabolism.

TF	CoCENA module affiliation	TFBS			DE analysis			
		Maroon gene module	Steelblue gene module	Lightgreen gene module	NEO_ctrl_vs_AD_ctrl	NEO_LPS_vs_AD_LPS	NEO_LPS_vs_NEO_ctrl	AD_LPS_vs_AD_ctrl
E2F1	maroon	X	X	X	up	up	n.s.	n.s.
CUX1	none	X	X		n.s.	n.s.	n.s.	n.s.
E2F4	none	X	X		n.s.	n.s.	n.s.	n.s.
HMBOX1	none	X	X		n.s.	n.s.	n.s.	n.s.
TBP	none	X	X		n.s.	n.s.	n.s.	n.s.
MYB	maroon		X	X	up	up	n.s.	n.s.
STAT1	lightgreen		X	X	down	down	n.s.	n.s.
IRF4	lightgreen		X	X	n.s.	down	down	n.s.
IRF1	seagreen		X	X	n.s.	down	up	up
STAT3	seagreen		X	X	n.s.	down	n.s.	up
FLI1	steelblue		X	X	n.s.	n.s.	down	down
EP300	none		X	X	n.s.	n.s.	n.s.	n.s.
SPI1	none		X	X	n.s.	n.s.	n.s.	n.s.
SPIB	none		X	X	n.s.	n.s.	n.s.	n.s.
STAT6	none		X	X	n.s.	n.s.	n.s.	n.s.
ZEB1	none		X	X	n.s.	n.s.	n.s.	n.s.
