# Supplementary material for: Oxidative phosphorylation is a key feature of neonatal monocyte immunometabolism promoting myeloid differentiation after birth
Source: Nat Commun. 2025 Mar 6;16:2239. doi: 10.1038/s41467-025-57357-w (PMC11885822; doi:10.1038/s41467-025-57357-w)
Supplement: Supplementary file 2 — Description of Additional Supplementary Files [file 41467_2025_57357_MOESM2_ESM.pdf]

Description of Additional Supplementary Files

Supplementary Data 1: Provides supplementary information on i) Differential gene expression analysis, ii) hCoCena functional enrichment, and iii) hCoCena TFBS enrichment
